# Initial Drug Sensitivity and Vulnerability to Substance Use Disorders: A Review of Individual Influences

**DOI:** 10.3390/biology15131077

**Published:** 2026-07-05

**Authors:** Shaun Smith, Judith Grisel

**Affiliations:** Department of Psychology, Bucknell University, Lewisburg, PA 17837, USA; sls065@bucknell.edu

**Keywords:** initial drug sensitivity, subjective drug response, individual differences, developmental factors, translational models, reward and aversion, associative learning, genetic influences, addiction, sex differences

## Abstract

Individual differences in the sensitivity and response to drugs of abuse are reliably associated with later patterns of substance use and, for some, progression to a substance use disorder diagnosis. This review synthesizes human drug challenge, longitudinal, and translational animal evidence to characterize how early or initial sensitivity to drugs, shaped by heritable and developmental factors, may relate to subsequent risk. These initial responses, through associative learning, may influence how drug-paired cues acquire motivational significance over time. Ample evidence suggests that early response variability is a biologically grounded and measurable correlate of SUD vulnerability, detectable well before chronic neuroadaptation, with potential utility for targeted prevention and intervention efforts.

## Simple Summary

People differ substantially in their experience of addictive drugs the first time they use them. For some, early use is rewarding and pleasurable; for others it is uncomfortable or aversive. These differences reflect genetic, developmental, sex, and life influences. This review presents evidence from human and non-human animal studies, across multiple drug classes, to show how early differences in the response to drugs can help predict those who will develop a substance use disorder. Across alcohol, nicotine, cannabis, stimulants and opioids, a large body of research points toward a recurring pattern: individuals whose first experiences with a drug are more rewarding and less unpleasant are more likely to continue using and, over time, more likely to develop a disorder. Early drug responses also shape learning in ways that influence future motivation and behavior. The evidence suggests that vulnerability to substance use disorder is detectable well before long-term neurobiological changes develop and that better understanding of these predisposing influences will inform more targeted prevention and intervention efforts.

## 1. Introduction

### 1.1. Definition and Scope

There are substantial individual differences in sensitivity and response to addictive drugs. These differences reflect genetic predispositions, developmental history, and the ecological context in which use first occurs. As a result, an initial exposure to a particular drug and equivalent dose may be experienced as rewarding, neutral, or aversive. Early effects may include euphoria or dysphoria, stimulation or sedation, anxiolysis or anxiogenesis, and antiemetic or emetic responses, among others. The pattern and relative weighting of these early responses contribute to the pattern of subsequent use and the liability for disordered use.

**Figure 1:**
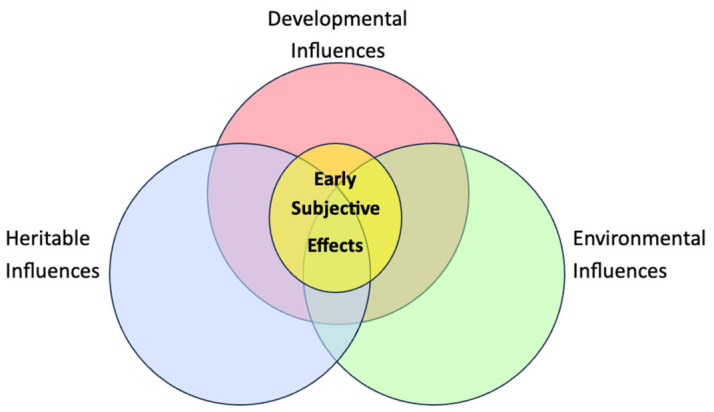
Early subjective experiences reflect heritable, environmental, and developmental influences that contribute to subjective states of both reward and aversion or influence the probability of developing disordered use.

We use initial drug sensitivity throughout this review as an umbrella term for the constellation of subjective (e.g., self-reported euphoria, dysphoria, liking), physiological (e.g., hormonal, cardiovascular, hypothermic), and behavioral (e.g., locomotor activity, place preference, self-administration) responses observed at or near the first drug experience as a product of heritable, environmental, and developmental factors (see Figure 1).

**Figure 2:**
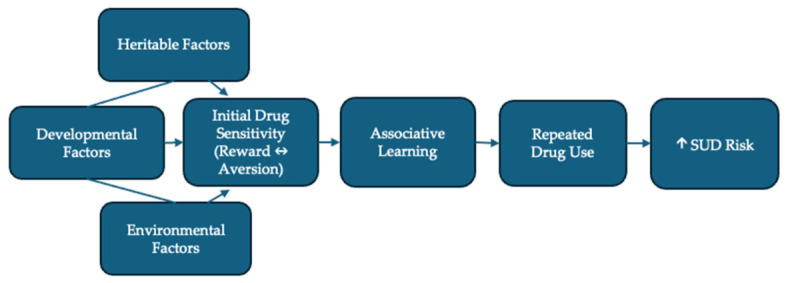
Conceptual pathway linking individual differences in initial drug sensitivity to substance use disorder vulnerability. Heritable and environmental influences often converge through developmental timing to jointly shape initial drug sensitivity, encompassing rewarding and aversive subjective, behavioral, and physiological responses. These early responses may influence associative learning and expectancy formation, which inform patterns of repeated drug use and ultimately contribute to vulnerability to substance use disorders. *Note.* ↑ indicates increased substance use disorder (SUD) risk.

We review subjective responses, dependent on self-report and the primary outcome in human laboratory and retrospective designs, and behavioral responses, which are observable directly and the primary outcome in translational animal models. Within this framework, reward and aversion are treated as independent dimensions that combine to significantly inform an individual’s net initial response to affect addiction liability (Figure 2).

### 1.2. Global Burden and Public Health Stakes

Substance use disorder (SUD) is defined clinically by patterns of use that result in significant impairment or distress, as operationalized by established diagnostic frameworks outlined in the Diagnostic and Statistical Manual of Mental Disorders, Fifth Edition [1] and the International Classification of Diseases, Eleventh Revision [2]. These frameworks converge on criteria including impaired control over use, continued use despite adverse consequences, tolerance and withdrawal, and disruption to social and occupational functioning. While these definitions are categorical for a diagnosis, the processes that give rise to SUDs unfold along a continuum of risk.

Although only a minority of users develop a SUD, the consequences of disordered use are substantial. Worldwide, alcohol accounts for an estimated 2.6 million deaths annually (roughly 4.7% of all deaths) and about 400 million people living with alcohol use disorders (AUD) [3]. In parallel, non-alcohol psychoactive drugs (excluding tobacco/nicotine) account for roughly 600 thousand deaths per year; in 2021, approximately 296 million people used such drugs, with an estimated 39.5 million experiencing drug use disorders, which represents a 45% increase over 10 years [4]. Despite some regional declines, overall mortality and disease burden remain high, particularly in low- and middle-income settings [5], and a substantial proportion of individuals with SUD do not receive treatment [6]. These figures underscore the need to better identify the factors that predict SUD trajectories, including early sensitivity.

Epidemiological data indicate that the transition from first use to SUD varies substantially across drug classes in both likelihood and timing. Progression within the first year ranges from approximately 5.3% for alcohol to 13% for opioids, with intermediate rates for stimulants (12.2%), sedatives (9.6%), and cannabis (7.5%). Over time, the proportion of users developing a disorder also differs across substances, reaching 50.4% for stimulants, 46.6% for opioids, 39% for sedatives, 37.5% for alcohol, and 34.1% for cannabis. Among those who develop a disorder, half of the cases occur approximately 2–4 years after onset, with shorter intervals for opioids and sedatives and longer intervals for alcohol [7]. Notably, some earlier estimates placed lifetime transition rates closer to 20–30% across substances [8]. Taken together, these figures suggest that while the proportion of users who progress to disorder varies by substance and method of estimation, the public health burden is substantial across all classes and elucidating critical factors may help in prevention and intervention efforts.

### 1.3. What This Review Covers

Across disciplines and methods, available evidence suggests that early response variability matters, that it is meaningfully related to later patterns of use, and that it warrants more direct experimental and prospective study. The sections that follow address three interrelated objectives. First, we examine the experimental designs used to capture first or early drug response in humans and animal models. Then we survey the biological, developmental, genetic, and environmental factors that contribute to individual differences in response across substances. Third, we analyze associative learning as a plausible mechanistic bridge between these initial sensitivities and longitudinal vulnerability, focusing on the distinct roles of reward-driven reinforcement and aversive effects in shaping subsequent behavior.

## 2. Empirical Approaches to Initial Drug Sensitivity and Individual Differences

### 2.1. Scope of the Evidence

Targeted literature searches were conducted using PubMed and Google Scholar, using combinations of terms including initial drug sensitivity, subjective drug response, first use, drug-naïve, conditioned place preference, reward prediction error, and substance-specific terms (alcohol, nicotine, cannabis, stimulant, opioid) crossed with sensitivity, vulnerability, and risk. Searches were supplemented by reference mining of relevant empirical studies and review articles. As a narrative review, rather than a systematic review, article selection prioritized methodological rigor, relevance to initial or early drug response, and representativeness across drug classes and study designs rather than exhaustive retrieval.

Given ethical and practical constraints, especially for illicit substances, direct observation of true first-exposure drug effects in humans is rare. As a result, the literature operationalizes initial drug sensitivity through a limited set of scientific approaches: controlled human laboratory paradigms, retrospective and prospective first-experience reports, longitudinal developmental designs, and translational animal models. Throughout this review, terms such as “predicts”, “is associated with”, and “contributes to” reflect statistically significant links established by the empirical research. As noted, the majority of human findings are observational, retrospective, or correlational; where shared genetic or environmental liability is a plausible alternative explanation for an observed association, we note this explicitly.

### 2.2. Controlled Human Laboratory Paradigms

Controlled human challenge studies provide the most precise method for isolating acute drug responses under standardized conditions. In these designs, participants receive a fixed or weight-adjusted dose of a substance, with pharmacokinetic variables such as blood concentration and timing tightly controlled.

Acute response is typically assessed using multidimensional subjective and physiological measures. Subjective effects are quantified using validated scales such as the Biphasic Alcohol Effects Scale (BAES), Subjective High Assessment Scale (SHAS), and Drug Effects Questionnaire (DEQ), capturing domains including stimulation, sedation, intoxication, liking, and wanting. Physiological and behavioral indices may include body sway, hormonal responses (e.g., cortisol, prolactin), and psychomotor performance.

Alcohol challenge paradigms represent the most extensively developed application of this approach [9,10,11,12,13]. These designs allow for the characterization of acute pharmacodynamic response independent of prior use history or learned associations and support the treatment of initial sensitivity as a multidimensional phenotype rather than a single global construct.

### 2.3. Retrospective and Prospective First-Experience Reports

A second major approach relies on self-reported accounts of initial drug experiences. These data are typically collected either retrospectively, through structured interviews or questionnaires assessing first-use reactions, or prospectively within longitudinal cohorts that capture early experiences closer to the time of exposure. Throughout, we use first exposure to refer to either a drug-naïve pharmacological encounter, primarily captured in animal models, or, more commonly in human studies, a recalled or prospective instance of early use within a defined developmental window.

Frequently studied measures in humans assess subjective reactions such as liking, relaxation, dizziness, nausea, stimulation, and aversion. Seminal work from Haertzen and colleagues demonstrated that individuals can reliably report qualitative features of their first drug experiences across substances [14,15].

Although retrospective reports are subject to recall bias and lack experimental control, they remain one of the few approaches capable of capturing literal first-use experiences across multiple drug classes [16]. As molecular imaging studies provide evidence that subjective drug effects correspond to measurable neurobiological processes [17,18], such findings support the interpretation that subjective reports reflect underlying neurobiological activity in reward-related systems.

### 2.4. Longitudinal and Developmental Designs

Longitudinal studies extend early-response research by linking initial subjective effects to subsequent patterns of use over time. In these designs, early experiences are assessed during adolescent or early use periods, and participants are followed developmentally.

Analytical approaches often include latent growth modeling, latent transition analysis, and repeated-measures frameworks to track changes in substance use behavior and symptom severity. These designs allow for the characterization of developmental trajectories while accounting for individual differences in early response. Examples include large cohort studies and clinical follow-ups that assess subjective effects alongside behavioral outcomes across adolescence and adulthood [19,20,21,22]. Although these approaches do not isolate pharmacological effects as precisely as laboratory paradigms, they may provide essential information about how early responses are embedded within development.

### 2.5. Translational Animal Models of Initial Response

At present, the clearest evidence for drug-naive sensitivity and its behavioral consequences comes from translational animal models, where first exposure and later trajectories can be experimentally investigated. Animal models provide a controlled framework for isolating early drug effects without confounds of expectation, prior experience, or self-report. Because subjective experience cannot be directly measured, reward and aversion are inferred through behavioral and physiological indices.

Animal models evaluate initial drug sensitivity at multiple levels, including subjective experience, physiological response, and behavior, under experimental conditions. While each approach captures different aspects of early response along with distinct limitations, together they define the primary methodological framework through which individual differences in initial drug sensitivity have been studied.

Several paradigms are commonly used:

- Conditioned Place Preference/Aversion (CPP; CPA) and Single-Exposure CPP (SE-CPP)

Measure associative learning between a drug and environmental context, indexing reward or aversion following one or more exposures [23,24,25]. Representative empirical studies include alcohol [26,27,28], cannabis/Δ9-tetrahydrocannabinol (THC) [29,30], stimulants [31,32,33], opiates and opioids [34,35].

- Intracranial Self-Stimulation (ICSS)

Assesses changes in reward threshold, providing a direct measure of drug-induced modulation of brain reward systems [36,37,38,39]. Representative empirical studies include alcohol [40,41,42,43], cannabis/THC [44,45], stimulants [46,47,48], and opioids [49,50]. Self-Administration (early acquisition phase)

Evaluates the reinforcing properties of a drug by measuring voluntary intake behavior [51,52]. Representative empirical studies include alcohol [53], cannabis/THC [54], stimulants [55,56,57], opioids [58].

- Pharmacological sensitivity assays

Include measures such as locomotor activity, analgesia, hypothermia, and taste reactivity, which index drug effects across multiple physiological systems. More broadly, baseline reward sensitivity can also be assessed using non-drug reinforcers. For example, Brennan and colleagues [59] identified individual differences in sucrose consumption and motivation, demonstrating that variability in reward sensitivity can be detected behaviorally and modulated by dopaminergic and opioidergic systems. Reviews of rodent alcohol research further describe the use of place conditioning, taste conditioning, and palatability assays as complementary measures of early response [60].

These models allow for precise manipulation of genetic background, sex, and environmental conditions, making them especially useful for isolating mechanisms underlying variability in initial drug response that might be conserved in humans.

## 3. Biological and Genetic Substrates of Initial Response

The clearest demonstration of an early-response phenotype comes from alcohol research. Early controlled alcohol challenge studies by Schuckit [9,10] evaluated young men with a family history of alcohol use disorder who consistently reported lower subjective intoxication at comparable blood alcohol concentrations (BAC) than matched controls. These early studies demonstrated that variability in acute alcohol response is detectable before chronic use or tolerance. Subsequent work [12,61,62,63] extended this observation, indicating that alcohol response is best understood as a multidimensional phenotype spanning stimulation, sedation, intoxication, and impairment (summarized in Table 1).

Within the multidimensional phenotype framework, risk is not solely defined by reduced response but rather by a pattern in which rewarding or stimulant effects are relatively enhanced, while sedative or aversive effects are diminished, shifting the balance of early experience toward reinforcement. Consistent with this differentiation, prospective alcohol-challenge work shows that greater stimulation, liking, and wanting, along with lower sedation, predict subsequent binge drinking and the accumulation of AUD symptoms over a multi-year follow-up [64,65,66], indicating that specific early response dimensions, rather than a single global response, track later risk. Genetic studies corroborate this framework. For example, allelic variation in GRIK1 and GABRA2 is thought to moderate subjective responses to alcohol, with carriers of specific alleles reporting reduced negative effects during controlled alcohol administration, a pattern associated with an increased vulnerability to alcohol use disorder [79,80,81].

Table 2 provides examples of studies linking early sensitivity to specific mechanisms. For example, dopaminergic signaling provides another central mechanism through which acute drug effects are translated into subjective experience. In human molecular imaging studies, the magnitude of self-reported stimulant effects corresponded to measurable changes in dopamine signaling. The subjective cocaine “high” increased with dopamine transporter (DAT) occupancy, and stimulant-like drugs were not reliably perceived until transporter blockade reached a substantial threshold [17,18]. In a related study, Volkow and colleagues [82] demonstrated that individuals with lower striatal D2 receptor availability showed greater reinforcing responses to methylphenidate, indicating that baseline receptor architecture constrains the intensity of acute drug reward. These findings establish that early subjective drug effects may be anchored in quantifiable neurochemical variation, lending biological validity to self-report as a measure of individual differences in acute drug response.

Another well-established body of evidence links genetic variation in alcohol metabolism genes to subjective response and subsequent risk for AUD. Polymorphisms in ADH1B and ALDH2 alter the rate at which ethanol is converted to acetaldehyde and subsequently cleared, producing substantial differences in acute physiological and subjective response. Individuals carrying variants that promote acetaldehyde accumulation often experience flushing, nausea, and discomfort at low doses, effectively shifting the initial experience toward aversion [83,84,85,86]. These pharmacokinetic differences impose a biological constraint on continued use: initial sensitivity is shaped not only by central nervous system response but also by peripheral metabolic processes that shift the hedonic valence of first exposure toward aversion.

Translational animal models provide a direct view of initial reward processes, isolating first-exposure effects from later adaptations. In a single-exposure conditioned place preference paradigm [27], both males and females from two inbred strains and one outbred strain exposed once to a moderate dose of alcohol showed robust conditioned place preference to the context associated with the drug. In rats, Nentwig et al. (2017) [28] performed a single ethanol pairing that produced place preference and revealed sex-dependent differences in expression depending on conditioning day and novelty of first apparatus exposure.

In another single-exposure design, Runegaard and colleagues [33] showed that a single exposure to cocaine was also sufficient to induce place preference in mice, with subsequent extinction and reinstatement procedures demonstrating how rapidly associative learning can follow initial reward. The single-exposure conditioned place preference model may be useful for elucidating particular genetic and environmental contributions to subjective reward or aversion. For example, early maternal separation, a model of developmental adversity, enhanced the initial rewarding effects of alcohol in male, but not female, transgenic mice deficient in the opioid peptide β-endorphin [117].

Intracellular signaling pathways can also modulate the balance between reward and aversion during early exposure. DiNieri and colleagues [87] showed that reducing cAMP response element-binding protein (CREB) activity in mice increased sensitivity to cocaine reward by diminishing sensitivity to dysphoric effects of the drug, as measured by intracranial self-stimulation. This shift led to a reward-dominant response profile, illustrating that variation downstream of receptor activation can affect how a drug is experienced.

Evidence from nicotine shows that early subjective response can be heritable and predictive even when the response is not strictly considered pleasant or pleasurable. Tsuang and colleagues [118] reviewed findings from the Vietnam Era Twin Registry, where nicotine dependence showed substantial shared genetic liability with alcohol dependence. Haberstick et al. (2011) [67] examined retrospective reports of initial cigarette reactions in 2482 young adult twins and siblings from the Add Health study using the Early Smoking Experience questionnaire. Positive initial experiences showed moderate heritability, as did an overall hedonic response and dizziness; multivariate modeling further identified a moderately heritable latent sensitivity factor that loaded most strongly on dizziness. In other words, a dizziness phenotype predicted risk for nicotine addiction. DiFranza et al. (2004) [68] followed adolescents who had inhaled cigarette smoke and found that relaxation after the first inhaled cigarette was a strong predictor of later dependence, while dizziness and nausea also independently predicted dependence symptoms. In contrast, irritation appeared to protect against subsequent risk.

More recently, Courtney et al. (2025) [69] examined early adolescents in the Adolescent Brain Cognitive Development (ABCD) study and found that both pleasant and unpleasant responses to first e-cigarette use predicted later e-cigarette use in adolescence. Despite sensations such as dizziness and nausea being ordinarily considered aversive, they predict both higher use and stronger dependence. Thus, dizziness may function as a proxy for heightened acute pharmacological sensitivity to nicotine such that individuals who register a stronger overall pharmacodynamic response may experience both rewarding and dizziness-related outcomes more intensely.

Similarly, early cannabis response varies substantially across individuals. In both community and clinical samples of adolescents and young adults, Zeiger and colleagues [72] found retrospective reports of cannabis’s subjective effects clustered into positive effects, such as relaxation and euphoria, and negative effects, such as dizziness, nausea and impaired control. Both types of effects were associated with cannabis dependence, although negative effects showed less consistent associations with recent use patterns. It remains unclear whether negative cannabis-related sensations reflect a truly aversive, hedonically negative experience or instead, as with nicotine-related dizziness, index heightened pharmacodynamic sensitivity to THC that manifests along an unpleasant dimension. On the other hand, longitudinal evidence from the Christchurch Health and Development Study found that among participants who used cannabis before age 16 [73], positive early reactions predicted cannabis dependence by age 21, while negative early reactions were not associated with later dependence.

Preclinical research shows that initial THC response differs systematically across genetic backgrounds. Parks and colleagues [93] examined acute THC response in the BXD recombinant inbred mouse panel and found heritable variation in locomotor suppression, hypothermia, and antinociception following initial exposure. These initial sensitivity measures to THC were associated with BXD phenotypes for cocaine, ethanol, and morphine response, indicating that early sensitivity to cannabinoids is informed by genetic variation that may affect responses to addictive drugs in general.

A large literature involving opioids demonstrates that early response varies across individuals along dimensions that include euphoria, activation, analgesia, and aversion, as well as physiological effects such as respiratory depression [102]. Biological differences in opioid sensitivity have been especially linked to genetic variation including polymorphisms in OPRM1, encoding the μ-opioid receptor [103]. The A118G polymorphism, in particular, can alter μ-opioid receptor structure, binding, and opioid sensitivity. Predictably, retrospective human reports indicate variability in the first subjective response. Bieber et al. (2008) [74] compared patients first exposed to prescription opioids for chronic pain who later did or did not develop prescription opioid use disorder (OUD) and found that the OUD group recalled stronger initial euphoric effects and more activating or stimulant-like effects. Morris et al. (2022) [75] reached a similar conclusion in a scoping review linking euphoric first-opioid response or drug liking with OUD or elevated OUD risk. Related placebo-controlled experiments by Bruehl and colleagues [119] found that higher recalled euphoric response during first medical opioid use predicted greater euphoria, less sedation, and greater desire to take morphine again during later laboratory morphine administration.

In a chronic-pain sample, Bruehl et al. (2015) [120] found that higher opioid-misuse risk was associated with greater desire to take morphine again, less negative subjective response, and greater morphine analgesia following morphine administration. Agrawal et al. (2022) [76] extended this finding to opioid misusers specifically, comparing individuals with and without OUD in pilot and replication samples. Across both samples, the OUD group retrospectively endorsed greater euphoria, activation, positive emotional effects, pruritus, and internalizing symptoms during initial opioid misuse. A twin study of double-blind alfentanil administration (Angst et al., 2012) [102] showed significant heritability for respiratory depression, nausea, and drug disliking, with familial effects for sedation, pruritus, dizziness and drug liking. These findings indicate that opioid sensitivity also includes both reinforcing and aversive dimensions, with the strongest evidence linking euphoric and activating early opioid responses with later OUD risk.

Corroborating preclinical work from Kallupi and colleagues [77] evaluated the relationship between initial analgesic response to oxycodone and subsequent extended-access self-administration in a population of genetically diverse rats. Greater initial analgesia predicted faster escalation of intake and higher addiction index scores.

In sum, across drug classes, early subjective responses differ in their balance of rewarding and aversive effects, with more positively valenced responses generally associated with continued use and risk for dependence while aversive effects generally constrain progression, although the strength of this relationship varies by substance and outcome [16]. Genetically informative multivariate twin studies show that SUD liability reflects both shared and substance-specific influences, including common vulnerability across illicit drug classes and disorder-specific genetic risk for alcohol and drug abuse/dependence [118,121]. Context further shapes how early risk is expressed: in adolescents, reward-related temperament and low effortful control interacted with peer alcohol use and parent-permitted sipping to predict heavier alcohol use and later AUD symptoms [20], while early life stress and parental risk for mood/SUDs were associated with greater positive subjective alcohol response under cue-rich drinking conditions [113].

## 4. Development, Sex, and Age-Dependent Modulation of Initial Response

In addition to heritable biological influences, initial drug sensitivity is also shaped by developmental timing and, in some cases, by prior non-volitional exposures such as prenatal drug exposure. Development may alter how receptor, signaling, and metabolic mechanisms are expressed, such that the same pharmacological exposure may produce different subjective and behavioral effects in individuals depending on when it occurs.

Across species, adolescence is associated with a shift in the balance between reward and aversion that directly alters early drug experience. In a comprehensive synthesis of human and animal work, Spear (2011) [111] describes adolescence as a developmental period in which reward-seeking behaviors are heightened while sensitivity to aversive consequences is reduced. Doremus-Fitzwater et al. (2010) [112] extend this observation to drug-specific effects, showing that adolescent organisms often exhibit enhanced sensitivity to the rewarding properties of substances alongside attenuated responses to aversive or impairing effects. This asymmetry is expressed at the level of initial experience, where early exposures are more likely to register as reinforcing and less likely to be hampered by sedation, dysphoria, or punishment. As a result, the impetus for repeat use is enhanced at a stage when behavioral regulation is still developing. Consistent with this, in a longitudinal sample of adolescents at elevated risk for substance use, Gresko et al. (2023) [21] found that subjective effects assessed during adolescence predicted general SUD severity across both legal and illegal substances into adulthood. Notably, both positive and negative early subjective responses were associated with greater severity, suggesting heightened motivational significance of early drug experiences in higher-risk populations.

In a longitudinal community sample, Scalco et al. (2021) [20] examined how temperament and social context jointly shape alcohol use during adolescence. Individual differences in self-regulation and approach-related behavior interact with peer use and parental permission to influence increases in alcohol use over time. These findings indicate that early alcohol experiences are shaped by preexisting motivational and regulatory factors, which influence how those experiences are encoded and carried forward into subsequent behavior.

In some cases, modulation of initial response begins prior to any voluntary use. For example, experimental work in rodents demonstrates that late-gestation exposure to ethanol alters postnatal responsiveness to alcohol-related cues. Chotro and Molina (1990) [105] showed prenatal exposure to ethanol in the amniotic fluid was sufficient to increase later odor preference and consumption. Likewise, Faas and colleagues [106] found that human newborns with prenatal alcohol exposure exhibited greater appetitive responses to alcohol odor, with response magnitude increasing as a function of maternal alcohol use. Lees et al. (2020) [107] reported that prenatal alcohol exposure was associated with increased likelihood of alcohol sipping in children ages 9 to 10, again, with evidence of dose-dependent effects. These findings suggest that what is often characterized as an initial response may, in some cases, reflect prior conditioning that shifts the hedonic value of the drug before first voluntary use.

Developmental timing may also contribute to how genetic risk factors are expressed. In a large meta-analysis of 43 datasets, Hartz et al. (2012) [88] found that early onset of regular smoking as well as nicotinic receptor variation (CHRNA5 rs16969968) each predicted heavier smoking. However, the effect of the risk allele was stronger among individuals who began smoking at age 16 or younger than among those who initiated later, and those with two risk alleles showed an even stronger effect. The variant itself was not associated with age at onset.

There is growing evidence of sex differences in early or initial drug sensitivity, but the literature suggests a complicated picture, depending on which substance and outcomes are assessed. For example, Cooper and Haney (2014) [95] found that women reported higher ratings of positive subjective effects from cannabis than men, but comparable intoxication and cardiovascular responses. In both human and non-human animal studies, elevated estradiol has been associated with enhanced positive subjective effects and increased sensitivity to the reinforcing properties of some drugs, but there is less research on androgens or progesterone. Several reviews [96,97,98,122] describe how hormonal fluctuations influence dopaminergic signaling within mesolimbic reward circuitry. For example, Parks et al. (2020, 2021) [93,94] showed sex differences in initial THC sensitivity across mouse strains, a finding also seen in human laboratory work showing that women report greater THC-induced subjective high at low doses while men report greater effects at higher doses [99]. Women have also been shown to experience a greater overall self-reported high from a single oral THC dose compared to men [100], though no sex differences were seen in psychotomimetic effects, heart rate, or verbal learning. Additionally, the CYP450 genotype has been shown to interact with sex to shape early subjective cannabis effects, with male slow metabolizers reporting more negative initial responses than female slow metabolizers [101].

The sex difference literature for stimulants is arguably the most consistent and replicable. Research in both humans and animals suggests that women may be more vulnerable to the reinforcing effects of stimulants, with estrogen possibly being one factor for this increased sensitivity [122]. In a 2001 study [123], Becker and colleagues showed that estradiol enhances cocaine- and amphetamine-induced behaviors in female rats, including acute locomotor activation and associated dopaminergic responses. Subsequent work demonstrated that females acquire stimulant self-administration more rapidly and show enhanced drug-seeking behavior during high-estradiol states, which are associated with greater subjective stimulant effects and increased dopaminergic response [96]. Torres et al. (2022) [98] further described how estrogen receptor signaling influences intracellular pathways relevant to psychostimulant reward. This body of work illustrates that early stimulant responses may be modified by sex hormones, perhaps to amplify reward sensitivity from the outset, but it also suggests that more research is needed in this important area.

Beyond developmental and physiological moderators, early environmental adversity may influence initial drug sensitivity. One idea is that initial exposures in children subject to early adversity may be particularly appreciated for their negatively reinforcing effects, that is, to dampen elevated states of anxiety or depression, and therefore are more likely to be repeated. Longitudinal evidence indicates that early-life stress is not only associated with substance use but with specific trajectories of escalation and co-use that imply differences in how early drug experiences are processed. In a large prospective cohort, Davis et al. (2023) [22] used latent transition analysis to examine how childhood adversity predicts transitions to alcohol and cannabis co-use. Individuals exposed to high levels of early adversity were more likely to transition into trajectories characterized by chronic and increasing co-use, with additional moderation by sex and internalizing symptoms. These findings demonstrate that early adversity is associated with distinct developmental influences, supporting the idea that initial drug effects are processed differently in individuals with prior stress exposure. Converging mechanistic evidence supports this interpretation. In a human molecular imaging study, Oswald et al. (2014) [114] found that individuals with greater histories of childhood adversity exhibited enhanced ventral striatal dopamine release in response to amphetamine administration. Thus, adversity would appear to affect reward properties of the drug, shifting the magnitude of acute subjective experience. However, this relationship may not be uniform across substances or consistent in direction. Carlyle et al. (2024) [115] conducted a secondary analysis of three randomized, placebo-controlled studies in which healthy volunteers received methamphetamine, d-amphetamine, or buprenorphine. Greater childhood adversity was associated with dampened subjective responses to stimulant drugs, specifically reduced ratings of feeling the drug, liking, and feeling high, with no significant effect on buprenorphine responses. These findings suggest that the impact of early adversity on initial drug sensitivity varies by substance and warrants further investigation.

Taken together, these findings indicate that age, sex, childhood adversity, prenatal exposure, and early social context may contribute to shaping how drug effects are experienced at first contact, in some cases amplifying reward-related signaling and biasing trajectories toward escalation (Table 2). Across these domains, developmental stage, physiological state, and genetic background alter the expression of relevant biological systems before first drug exposure. By the time an individual first encounters a substance, reward, aversion, and regulatory systems may already differ substantially as a function of age, experience, and internal state, producing meaningful variation in early subjective response before long-term neuroadaptations occur and shaping the likelihood of subsequent use and adaptation.

## 5. Analyzing the Relationship Between Early Response and Longitudinal Vulnerability

Early differences in sensitivity to drugs of abuse are often linked to later behavior through associative learning processes that provide a bridge between initial subjective response and subsequent substance-related behavior. Initial rewarding and aversive effects of drugs shape how rapidly drug-paired cues and contexts acquire motivational significance. Early response variability functions as a learning signal that occurs prior to the development of chronic tolerance or withdrawal-driven habit systems.

As we have indicated, individual differences in early drug response can be organized along two primary dimensions: reward-driven reinforcement and aversive-driven inhibition. However, these processes may co-occur within the same initial exposure as partially independent signals that differentially influence subsequent behavior. Early drug experiences are therefore not uniformly positive or negative: substances may produce simultaneous rewarding and aversive interoceptive effects, such as relaxation alongside nausea or dizziness following nicotine [68] and stimulation alongside dysphoria with cocaine [124].

Solomon and Corbit’s Opponent-Process Theory [125] framed the acute hedonic response as the trigger for a secondary opponent affective process, providing an early account of how initial drug effects set conditions for later behavioral adaptation. Contemporary reinforcement learning models build on this foundation, suggesting that an initial drug response generates a reward prediction error (RPE) mediated by mesolimbic dopamine to update the brain’s expected value of the drug and its associated context [126]. The magnitude of this signal at first exposure sets the starting conditions for how rapidly and strongly associative learning proceeds. Incentive sensitization theory [127] extends this account by distinguishing between the hedonic impact of a drug (“liking”) and the motivational value attributed to drug-paired cues (“wanting”). These frameworks generate an account of how early response variability translates into individual differences in long-term cue-driven behavior. A stronger initial RPE promotes faster cue-drug association, and repeated drug-paired cue exposure can amplify wanting independent of changes in liking. The sections that follow address how reward and aversive components of early response each contribute to this process.

### 5.1. Reward

A core mechanism through which early sensitivity is thought to exert its influence is reinforcement learning supported by mesocorticolimbic dopamine signaling. Applied here, the reinforcement learning framework offers a mechanistic account of why stronger early stimulation or euphoria and weaker competing aversive effects bias subsequent associative learning toward drug-paired cues and contexts.

**Figure 3:**
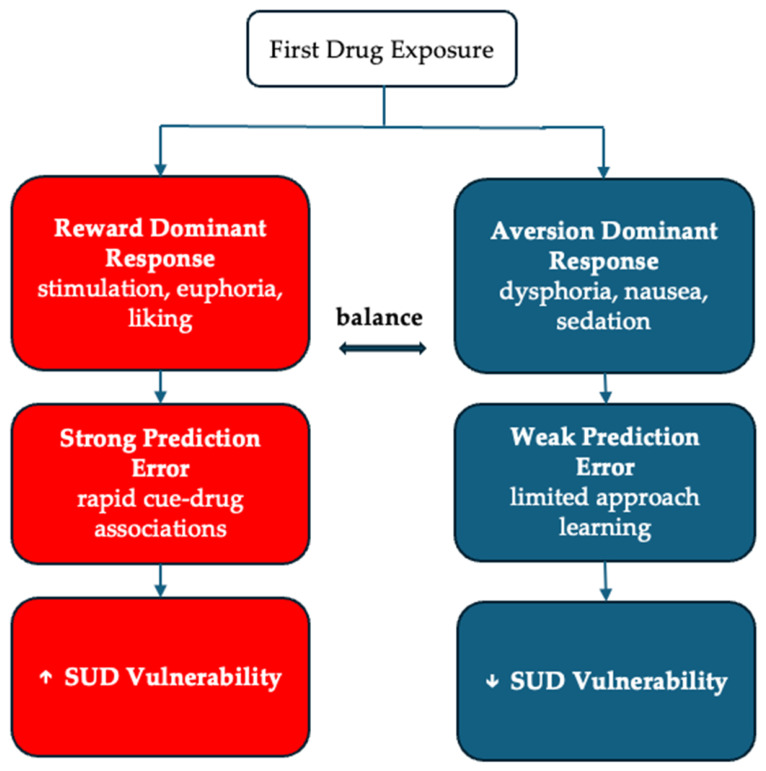
Reward-aversion balance framework. At first drug exposure, the relative strength of reward-dominant responses (stimulation, euphoria, liking) versus aversion-dominant responses (dysphoria, nausea, sedation) sets the size and direction of the prediction error, the gap between the expected and actual drug effect that drives the brain to update value and form new associations. A strong, positive prediction error favors rapid cue-drug learning and is linked to escalating use. A weak or negative prediction error instead limits cue-drug learning and may suppress continued use. *Note.* ↑ indicates increased SUD vulnerability; ↓ indicates decreased SUD vulnerability.

Evidence from Flagel and colleagues using animal models [128,129] provides a behavioral analog for this theoretical distinction through the sign-tracker (ST) versus goal-tracker (GT) phenotype. In Pavlovian auto-shaping paradigms, sign-trackers preferentially attribute incentive salience to the reward-predictive cue (the “sign”) itself, interacting with the cue as a motivational magnet. STs are also more likely to escalate their drug use. In contrast, goal-trackers treat the cue as a mere predictor, approaching the reward location (the “goal”) instead. Notably, these divergent learning outcomes emerge rapidly; Flagel et al. (2007) [130] observed that STs exhibit greater expression of dopamine D1 receptor mRNA after a single training session, whereas GTs eventually show higher levels of tyrosine hydroxylase and D2 receptor mRNA. These findings indicate that the ST phenotype reflects a specific vulnerability toward cue-driven behavior and higher drug intake, providing an empirical basis for how early sensitivity can bias associative learning processes toward cue dominance over time (Figure 3). Although the ST and GT distinction offers a compelling mechanistic account, it derives entirely from rodent auto-shaping procedures, and the degree to which it maps onto comparable individual differences in human cue reactivity, as opposed to simply running in parallel with them, remains an important empirical question.

These phenotypes are particularly relevant to initial sensitivity because the divergence between STs and GTs emerges rapidly after minimal drug exposure, suggesting that individual differences in how early reward signals are processed can quickly translate into distinct cue-learning trajectories. This trajectory is consistent with later-stage neuroimaging evidence: Huang et al. (2024) [131] found that individuals with heroin use disorder showed significantly greater cortico-striatal activation in response to drug cues compared to both neutral cues and food cues and that this hyperreactivity also correlated with craving. Findings such as these illustrate how early cue-learning, over time, produces the biased motivational salience that defines established addiction.

### 5.2. Aversive

Within the same framework, individuals whose initial responses are dominated by aversive effects, including dysphoria, nausea, sedation, generate weaker or negative prediction errors, limiting the acquisition of approach-oriented associations. Aversive effects can coexist with reinforcing signals within the same exposure [68,124], and their relative balance shapes the net learning signal.

The dynorphin/kappa opioid receptor system provides one candidate mechanism through which aversive components are expressed at the neurobiological level. Preclinical work summarized by Cayir et al. (2024) [132] suggests that kappa-opioid receptor activation can reduce self-administration and conditioned place preference by producing dysphoric or aversive-like effects. Like dopaminergic and mu-opioid signaling, kappa opioid system activity is likely to vary across and within individuals, contributing to differences in how aversive drug effects are experienced during initial exposure [133].

Further work on reward prediction and aversion circuitry by Graziane (2018) [134] highlights the role of the lateral habenula in encoding negative prediction errors and suppressing dopaminergic signaling when outcomes are worse than expected, providing a neural substrate through which aversive drug effects can counterbalance reward-driven learning. Within this framework, early drug experiences that recruit habenula-mediated signaling may limit or reverse reinforcement by reducing dopamine-driven value updating, particularly when initial exposure produces mixed or rapidly shifting affective responses.

Powers et al. (2024) [78] provide a useful example of how aversive valuation can alter early cocaine-taking trajectories. Importantly, this model differs from prior self-administration paradigms by introducing an aversive stimulus at the onset of drug exposure rather than after stable intake is established. In this study, rats self-administered intravenous cocaine paired with intraoral quinine from the first session, allowing reward and aversion to be experienced together rather than introducing punishment after cocaine intake was established. Three groups emerged: one group showed very little cocaine intake, one escalated intake despite the aversive quinine cue, and a third showed high intake on the first day followed by a rapid decline across sessions. Notably, the escalating group continued to self-administer cocaine despite exhibiting aversive taste reactivity comparable to low-intake animals, indicating that persistence of use did not reflect reduced aversive sensitivity. The descending group later showed the greatest net aversive taste reactivity to quinine, and the steepness of the decline in cocaine intake predicted the strength of that aversive response. These findings suggest that early high intake does not necessarily reflect stable reward dominance. In some individuals, aversive effects may become strong enough within the first several exposures to suppress subsequent use. The authors further suggest that the initial high intake in this group may reflect novelty-driven engagement with the drug-cue pairing, while the subsequent decline may be driven by emerging aversive valuation, potentially amplified by delayed dysphoric effects of cocaine at higher doses.

## 6. Limitations

This review has several limitations that warrant consideration. First, most of the human evidence relies on retrospective or correlational designs rather than experimental manipulation of first exposure, which constrains causal inference. Apparent associations between early response and later SUD risk may partly reflect shared genetic or environmental liability that independently shapes both phenotypes, rather than a direct causal pathway from initial response to disorder. While family history and twin designs can help partition this variance, they cannot fully rule out confounding at the individual level. Related to this, what is often labeled first exposure in human research is typically a recalled instance of early use within a developmental window rather than a literal drug-naïve pharmacological exposure, since true first data can rarely be captured prospectively for ethical and practical reasons; this conflation should be kept in mind when comparing across study designs. Finally, because studies reporting significant early response and SUD associations are more likely to be published than null results, the synthesized literature may overrepresent positive findings such that the relatively few null or contradictory results identified here may not fully capture the mixed nature of the research landscape.

Second, translational inference between animal and human work carries its own constraints. Conditioned place preference, intracranial self-stimulation, and self-administration paradigms isolate reward and aversion with a precision unavailable in human research, but the degree to which a given rodent phenotype, such as the ST and GT distinction, maps onto human cue reactivity and SUD risk remains an inference rather than a demonstrated equivalence. Dose dependency further complicates this: although early response is widely assumed to scale with dose, this relationship is acknowledged inconsistently across the cited literature and has not been systematically incorporated into existing models of initial sensitivity.

Third, the depth of the evidence is uneven across drug classes. The alcohol literature benefits from a multi-decade tradition of controlled human challenge studies that has no real counterparts for opioids, where ethical and regulatory constraints on administering an abused substance to drug-naïve volunteers leave the evidence base more dependent on retrospective reports and preclinical models. The smaller number of studies focused on other drug classes should be considered when comparing the strength of conclusions across substances in this review.

## 7. Conclusions

Initial drug sensitivity reflects systematic variation in the interaction between drug pharmacology and individual biological and experiential factors at first exposure. Throughout alcohol challenge paradigms, retrospective and prospective first-use reports, translational animal models, and genetically informed designs, early responses emerge as structured individual differences that can foreshadow later patterns of use in ways that are biologically plausible, developmentally patterned, and often measurable well before neuroadaptation to chronic exposure drives behavior. The clearest human evidence remains strongest in alcohol, where controlled challenge studies have shown that lower subjective intoxication, or alternatively a profile characterized by greater stimulation and reward with lower sedation, predicts later binge drinking and alcohol use disorder symptoms. Across other drug classes, the same basic signals recur; early experiences that are more positively valenced, less constrained by aversive feedback, or differently weighted across reward and discomfort are often associated with a greater likelihood of continued use, escalation, or later disorder.

Early responses identify a critical stage of substance involvement that remains largely understudied. Understandably, much of addiction research has focused on the neuroadaptations, compulsive patterns, and cumulative harms that characterize later stages of the disorder. However, the extant research suggests that much of the liability shaping those later trajectories is visible earlier, at or near the first exposure. In that sense, initial sensitivity may offer critical insight into why similar exposures lead to markedly different trajectories across individuals. It is also at a point in the progression toward disorder where prevention may be especially fruitful. Better characterization of the factors that contribute to early response profiles may help distinguish individuals for whom prevention and early intervention efforts could be better tailored and, therefore, more efficacious.

Initial sensitivity may have practical value for prevention. Brief self-report instruments designed to capture early subjective responses, such as measures adapted from the Biphasic Alcohol Effects Scale (BAES) or structured first-use questionnaires modeled on the Early Smoking Experience measure, could potentially be incorporated into routine adolescent and young-adult health screening alongside family-history assessment. Such measures could help identify individuals whose early response profiles are associated with elevated risk before regular patterns of use become established. This approach would not require genetic testing or laboratory drug-challenge procedures and could build on primary-care, school-based, or collegiate health-screening infrastructure already used to assess related risk factors. Realizing this potential will require prospective validation showing that brief self-report measures of initial response provide predictive value beyond established risk factors such as family history and early age of onset and that screening can be implemented without inadvertently normalizing or encouraging early substance use.

Future clinical research will make the most progress through prospective designs that move observation closer to actual first or early use. Ecological momentary assessment (EMA) approaches appear especially promising here. When combined with repeated measurement of subjective effects, affective state, context, cue exposure, peer setting, and early use patterns, EMA-based designs could capture early human drug experiences with a level of temporal and ecological validity that retrospective reports cannot provide. In addition, combining EMA designs with genetic or other neurobiological predictors may aid in distinguishing pharmacologic sensitivity from influences of expectancy, setting, stress, and social context.

Assessments that integrate family history, adversity exposure, prenatal or early sensory exposure, sex, pubertal or hormonal status, and baseline reward-related states and traits will likely provide a more accurate assessment of initial sensitivity than studies focused solely on drug exposure. Future studies should therefore strive to place first or early use within a broad developmental history and social context. This will reflect the evidence showing that risk is often patterned by combinations of factors rather than single moderators in isolation.

The field would also benefit from more translational studies between human and non-human animal models. For example, the recent work using genetically diverse rodents [77] is especially promising because it captures initial-response variability at scale and links it to later addiction-like outcomes under controlled conditions. Studies of this kind provide a useful model for how we might go forward with systematic characterization of early sensitivity phenotypes. The translational task now is not merely to show that first-exposure reward or aversion exists but to determine which dimensions of that response are stable, which are context-dependent, and which most reliably forecast later trajectories across substances.

Animal models will remain especially important for addressing questions that cannot be answered ethically or practically in humans. These models permit more precise control over genetic background, developmental timing, drug dose, route of administration, environmental context, and prior exposure history, allowing investigators to isolate specific determinants of initial sensitivity and their causal relationship to later drug-related behaviors. Future work using genetically diverse populations, longitudinal designs and emerging tools for circuit- and cell-specific manipulation may help identify the neurobiological mechanisms linking early reward and aversion profiles to subsequent patterns of drug seeking, escalation, and addiction-like behavior. Such approaches will be particularly valuable for disentangling pharmacological sensitivity from expectancy, social influences, and other contextual factors that are difficult to separate in human populations.

In that sense, the main takeaway is that research on initial drug sensitivity is poised to help explain individual differences in the initiation, escalation, and perpetuation of substance use and abuse. The earliest subjective and behavioral effects of drugs appear to capture a ripe intersection of biology, development, environment, and learning before the complex sequelae associated with addictive states develop.

## Figures and Tables

**Table 1:** Predictors of initial drug response and associated outcomes.

Predictor (Initial Response Phenotype)	Operationalization/Model	Direction of Effect	Outcome	Source
Alcohol: low subjective intoxication/low response	Human alcohol challenge; family-history paradigm	↓ perceived intoxication at comparable BAC	↑ AUD liability	[9,10,11,12,61,62]
Alcohol: high stimulation/reward with low sedation	Human alcohol challenge; BAES; prospective follow-up	↑ stimulation, liking, wanting; ↓ sedation	↑ binge drinking → ↑ AUD symptoms	[63,64,65,66]
Alcohol/cocaine: single-exposure reward	CPP/SE-CPP animal models	↑ place preference after one exposure	Initial reward → cue/context learning	[27,28,33]
Nicotine: first-use relaxation, dizziness, nausea, pleasurable or unpleasant effects	First cigarette and first vaping studies; adolescent follow-up	Mixed reward + aversion profile	Early nicotine sensitivity → dependence symptoms/later vaping	[67,68,69,70,71]
Cannabis: positive early reactions	Prospective cohort and retrospective subjective-response studies	↑ liking, relaxation, euphoria, positive affect	↑ cannabis dependence risk	[72,73]
Opioids: euphoric/activating initial response	Retrospective first-response studies; case–control; scoping review	↑ euphoria, liking, activation, positive emotion	↑ OUD risk	[74,75,76]
Opioids: initial analgesic sensitivity	Prospective preclinical model before extended-access self-administration	↑ initial oxycodone analgesia	↑ addiction-index scores → faster escalation	[77]
Cocaine: reward competing with aversive valuation	Early self-administration with quinine-paired cocaine	High initial intake may ↑ or ↓ depending on aversion	Aversive valuation can redirect early cocaine trajectory	[78]
Cross-substance: positive/aversive first-use reactions	Retrospective and prospective first-experience literature	↑ positive response/↓ aversive constraint	↑ continued use; aversion may ↓ progression	[14,15,16]

*Note.* ↑ = increased or greater; ↓ = decreased or lower; → = leads to or is associated with progression toward. Symbols reflect the direction or nature of associations reported in the cited literature and do not imply causation unless otherwise stated in the source.

**Table 2:** Sources of individual differences in initial sensitivity across major drug classes.

Source of Variation	Substance	Mechanism/Factor	Effect on Initial Response	Source
Alcohol challenge phenotype	Alcohol	Family history; multidimensional acute response	↓ intoxication; altered stimulation/sedation balance	[9,10,11,61,62]
Metabolic genotype	Alcohol	ADH1B/ALDH2 variation; acetaldehyde accumulation	↑ flushing, nausea, discomfort, aversion	[83,84,85,86]
Subjective-response candidate genes	Alcohol	GABRA2/GRIK1 moderation of alcohol response	Altered negative, sedative, or reinforcing effects	[79,80,81]
Dopaminergic architecture	Stimulants	DAT occupancy; striatal D2 receptor availability	↑ or ↓ “high,” liking, reinforcement	[17,18,82]
Intracellular signaling	Cocaine	CREB-related modulation of reward and dysphoria	↑ cocaine reward; ↓ dysphoric constraint	[87]
Heritable early subjective response	Nicotine	Twin/sibling variation in first cigarette reactions	↑ heritable positive effects, dizziness, hedonic response	[67]
Developmental genetic moderation	Nicotine	CHRNA5 rs16969968 × early smoking onset	↑ genetic risk expression in early-onset smokers	[88]
Endocannabinoid system variation	Cannabis	CNR1/FAAH variation	↔ cannabis effect, reward, dependence vulnerability	[89,90,91,92]
Strain-dependent THC sensitivity	Cannabis	Genetic background in BXD and inbred mouse models	↔ hypothermia, locomotor suppression, antinociception, tolerance	[93,94]
Sex/hormonal modulation	Cannabis; stimulants	Ovarian hormones; sex-linked reinforcement sensitivity	↑ cannabinoid or stimulant reward sensitivity in some paradigms	[94,95,96,97,98,99,100,101]
Acute opioid heritability/familial effects	Opioids	Genetic and familial variation in alfentanil response	↑ heritable nausea, respiratory depression, disliking; familial liking/sedation	[102]
Opioid receptor variation	Opioids	OPRM1/μ-opioid receptor sensitivity	↔ opioid reward, analgesia, aversion	[103,104]
Prenatal alcohol exposure	Alcohol	Prenatal sensory/neurobiological exposure to ethanol	↑ alcohol odor preference, appetitive odor response, early sipping risk	[105,106,107]
Prenatal opioid exposure	Opioids/stimulants	Developmental opioid exposure altering later reward sensitivity	↑ morphine CPP, heroin/cocaine self-administration, methamphetamine sensitization	[108,109,110]
Adolescent developmental state	Multiple	Reward-aversion imbalance; receptor remodeling; regulatory immaturity	↑ reward sensitivity; ↓ aversive/impairing constraint	[111,112]
Stress/social context	Alcohol/cross-substance	Peer use, parental permission, early life stress, familial risk	↑/↔ positive response or escalation risk in vulnerable contexts; dampened stimulant response with higher adversity severity.	[20,22,93,113,114,115]
Premorbid neurobehavioral liability	Multiple	Reward temperament; effortful control; executive function; brain structure/function	Alters pre-exposure reward processing and regulatory control	[21,116]

*Note.* ↑ = increased or greater; ↓ = decreased or lower; ↔ = mixed or bidirectional effects across studies. Symbols reflect the direction or nature of associations reported in the cited literature and do not imply causation unless otherwise stated in the source.

## Data Availability

No new data were created or analyzed in this study. Data sharing is not applicable to this article.

## Abbreviations

The following abbreviations are used in this manuscript:

SUD	Substance Use Disorder
AUD	Alcohol Use Disorder
OUD	Opioid Use Disorder
CPP	Conditioned Place Preference
CPA	Conditioned Place Aversion
SE-CPP	Single-Exposure Conditioned Place Preference
ICSS	Intracranial Self-Stimulation
RPE	Reward Prediction Error
EMA	Ecological Momentary Assessment
BAES	Biphasic Alcohol Effects Scale
SHAS	Subjective High Assessment Scale
DEQ	Drug Effects Questionnaire
BAC	Blood Alcohol Concentration
DAT	Dopamine Transporter
THC	Δ9-Tetrahydrocannabinol
CREB	cAMP Response Element-Binding Protein
OPRM1	Opioid Receptor Mu 1
GRIK1	Glutamate Ionotropic Receptor Kainate Type Subunit 1
GABRA2	Gamma-Aminobutyric Acid Type A Receptor Subunit Alpha2
ADH1B	Alcohol Dehydrogenase 1B
ALDH2	Aldehyde Dehydrogenase 2
CHRNA5	Cholinergic Receptor Nicotinic Alpha 5 Subunit
CNR1	Cannabinoid Receptor 1
FAAH	Fatty Acid Amide Hydrolase
ST	Sign-Tracker
GT	Goal-Tracker
